# Use of Magnetic Field for Mitigating Gyroscope Errors for Indoor Pedestrian Positioning †

**DOI:** 10.3390/s18082592

**Published:** 2018-08-07

**Authors:** Ming Ma, Qian Song, Yang Gu, Zhimin Zhou

**Affiliations:** School of Electronic Science, National University of Defense Technology, Hunan 410073, China; danosong@126.com (Q.S.); gu_yang_1990@126.com (Y.G.); kdzhouzhim@163.com (Z.Z.)

**Keywords:** gyroscope bias, indoor pedestrian positioning, magnetometer calibration, quasi-static magnetic field

## Abstract

In the field of indoor pedestrian positioning, the improved Quasi-Static magnetic Field (iQSF) method has been proposed to estimate gyroscope biases in magnetically perturbed environments. However, this method is only effective when a person walks along straight-line paths. For other curved or more complex path patterns, the iQSF method would fail to detect the quasi-static magnetic field. To address this issue, a novel approach is developed for quasi-static magnetic field detection in foot-mounted Inertial Navigation System. The proposed method detects the quasi-static magnetic field using the rate of change in differences between the magnetically derived heading and the heading derived from gyroscope. In addition, to eliminate the distortions caused by system platforms and shoes, a magnetometer calibration method is developed and the calibration is transformed from three-dimensional to two-dimensional coordinate according to the motion model of a pedestrian. The experimental results demonstrate that the proposed method can provide superior performance in suppressing the heading errors with the comparison to iQSF method.

## 1. Introduction

High accuracy and infrastructure-free indoor pedestrian positioning is a highly desired ability for finding and rescuing firefighters or other emergency first responders, or for personal navigation assistance. With the adoption of Global Navigation Satellite System (GNSS), positioning in outdoor environment has witnessed a dramatic success. However, GNSS performs poorly in indoor environment and is not suitable for indoor positioning because of weak signal reception and radio wave multi-path effect. Developers have suggested various indoor positioning technologies such as Ultrasound [1,2], Radio frequency (RF) [3,4] and Ultra wide band (UWB) [5,6]. The Ultrasonic positioning system localize a pedestrian based on the propagation time of ultrasonic wave and it can achieve an accuracy within a meter. However, it needs large-scale basic infrastructures resulting in extra costs. The RF positioning technology is one of the most cost-effective methods because wireless local area network (WLAN) is available in most indoor scenarios. Nevertheless, due to the multipath arising from reflection and the scattering characteristic of indoor environments, RF do not achieve high levels of accuracy when applied to large areas [7]. The accuracy of most of the RF based positioning systems is typically in the range of a few meters. Wi-Fi fingerprinting positioning is another solution for indoor positioning. In this method, first of all, a radio map will be created by collecting fingerprints of a scene and then it will be used to estimate user location [8]. The radio map has to be acquired before positioning can be performed, which render Wi-Fi fingerprinting positioning inappropriate for emergency use.

A practical solution to indoor positioning is to use inertial sensors such as microelectromechanical systems (MEMS) gyroscopes and accelerometers. Inertial-based positioning systems can track the position of the user with no pre-installed infrastructures or a geographical fingerprint. These systems are generally in the form of wearable devices, which can be worn on the helmet, belt or shoe of a person [9,10]. Among such systems, the foot-mounted inertial measurement unit (IMU) is preferred in our system [11,12], because the foot-mounted IMU has stance phases, which can be used to suppress the long-term drifts of inertial sensors with the Zero-velocity updates (ZUPT) aided Extended Kalman Filter (EKF) method [13,14,15]. However, the ZUPT still suffer from modelling errors and measurement errors [16]. During the stance phases detected by Zero-velocity detection algorithms, the heading (yaw) and gyroscope bias cannot be observed from zero-velocity measurements. During motion phases, the short-term drift of inertial sensors cannot be compensated for and it accumulates slowly. The influence of these errors on the heading cannot be eliminated by the ZUPT algorithm [17].

In order to solve this problem, other aiding sensors or information sources are utilized. For example, with magnetometers, the measure of the Earth’s magnetic field can be used to estimate errors associated with gyroscopes. In Reference [18], the difference between the gyroscope-acquired heading and magnetically derived heading is utilized as the heading error, which was fed into the ZUPT aided EKF. However, the magnetic field suffers from severe interference in indoor environment, resulting in extra measurement errors. In Reference [19], a magnetic positioning system (MPS) was adopted to aid the dead-reckoning inertial navigation system (DR-INS). The range information provided by the MPS is used as the measurement fed into EKF to correct the position of the DR-INS. This method can provide bounded position errors of 1–2 m over significantly long periods of time up to 45 min. The shortcoming of the method in Reference [19] is that the magnetic field generating coils must be installed in advance before positioning can be performed. Moreover, this method might be not suitable for multi floor application due to the assumption that the field-generating coil and the receiving node lie in the same plane. In Reference [20,21,22], magnetic fingerprinting based positioning methods were developed. The magnetic readings acquired from magnetometer are compared with magnetic readings in the previously created database. User position is determined as the position in fingerprint database with the closest match to current magnetic measurements. Creating the fingerprint database is a time-consuming process, hence the magnetic fingerprinting method is not inappropriate for emergency use. In Reference [23], a Quasi-Static magnetic Field (QSF) approach was developed to estimate the heading error in perturbed indoor environments. The approach detects a QSF based on the rate of change in the magnetic field magnitude. Although this method can suppress the heading drift and improve the positioning accuracy, it can lead false-alarm detection when the magnitude of each axis changes with different ratio while the magnitude of total field has very slight changes. In that case, using the magnetic information may induce extra errors during the estimation of the gyroscope’s errors. In Reference [24], an improved Quasi-Static magnetic Field (iQSF) method is introduced, which detects the quasi-static field periods depending on the change rate of heading and the magnitude of the total magnetic field. Compared with the QSF method, the detection accuracy and robustness of the iQSF method are much better. The limitation of the iQSF method is that it can only take effect when a person walks along straight-line paths. If the trajectory is curved or more complex, the iQSF method will not work for most of the time.

To address the aforementioned issues, we propose a novel quasi-static magnetic field detection approach for foot-mounted IMU system. The proposed method detects the quasi-static magnetic field using the rate of change in differences between the magnetically derived heading and the heading derived from gyroscope. The proposed method will not be subject to the straight-line constraint. The quasi-static magnetic field detected by the proposed method can be utilized as the measurement, which can be fed into the ZUPT-aided EKF to estimate the gyroscope biases. Moreover, a calibration method of the magnetometer is proposed to eliminate the hard iron and soft iron distortions caused by system platform and shoes. The calibration of the magnetometer is carried out before it is adopted for positioning.

## 2. Magnetometer Calibration

### 2.1. Heading and Inclination Angle Estimation Using Magnetic Field

**Figure 1:**
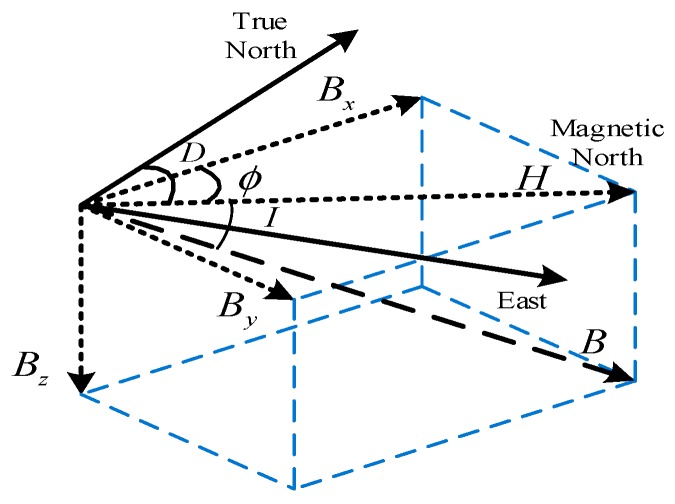
Magnetic field components and their relationship with true North.

The Earth’s magnetic field can be precisely measured and utilized to estimate heading by magnetometers. The three-dimensional Earth’s magnetic field vector is shown in Figure 1.

Here B denotes the total magnetic field, H is the horizontal component of B, D is the declination angle between H and the true north. $B_{x}$, $B_{y}$ and $B_{z}$ are the three orthogonal components of total magnetic field. The magnetically derived heading with respect to the true North is estimated as:

(1) $$\varphi =\phi \pm D=\tan^{-1}(\frac{B_{y}}{B_{x}})\pm D$$

Inclination angle I is given by

(2) $$I=\tan^{-1}(\frac{B_{z}}{\left\|H\right\|})$$

From Equation (1), it can be observed that the magnetic field components should be transformed to navigation frame (defined to be North-East-Down on the ground) so as to find the horizontal component of the Earth’s magnetic field. Let $\left(m_{x}^{k},\;m_{y}^{k},\;m_{z}^{k}\right)$ be the sample of the three-axis magnetometer’s measurement at time k in sensor body coordinate frame. Let $\left(r_{k},\;p_{k},\;y_{k}\right)$ be the Euler rotation angles between body coordinate and navigation frame, then the transformation can be expressed by roll ($r_{k}$) and pitch ($p_{k}$) angles:

(3) $$\left[\begin{array}{c} B_{x}\\ B_{y}\\ B_{z} \end{array}\right]=\left[\begin{array}{ccc} \cos p_{k} & 0 & -\sin p_{k}\\ 0 & 1 & 0\\ \sin p_{k} & 0 & \cos p_{k} \end{array}\right]\times \left[\begin{array}{ccc} 1 & 0 & 0\\ 0 & \cos r_{k} & \sin r_{k}\\ 0 & -\sin r_{k} & \cos r_{k} \end{array}\right]\times \left[\begin{array}{c} m_{x}^{k}\\ m_{y}^{k}\\ m_{z}^{k} \end{array}\right]$$

where $r_{k}$ and $p_{k}$ are obtained from the accelerometer measurement as follows:

(4) $$\begin{array}{l} p_{k}=-\sin^{-1}(a_{x}^{k}/g)\\ r_{k}=\sin^{-1}(a_{y}^{k}\times (g\times \cos p_{k})) \end{array}$$

where g denotes acceleration of gravity. Noting that the heading estimation proceeds only during the stance phase, when $r_{k}$ and $p_{k}$ in Equation (3) can describe the attitude of the sensors. During the stance phase, the heading $y_{k}$ is the only Euler rotation angle unknown. In addition, the magnetically derived heading φ is independent of the orthogonal field component $B_{z}$ according to Equation (1). Therefore, three-axis magnetometer’s measurement can be replaced by two-axis measurement and the replacement is given by

(5) $$\begin{array}{l} m_{x}^{k^{\prime }}=m_{x}^{k}\times \cos p_{k}+m_{y}^{k}\times \sin p_{k}\times \sin r_{k}-m_{z}^{k}\times \sin p_{k}\times \cos r_{k}\\ m_{y}^{k^{\prime }}=m_{y}^{k}\times \cos r_{k}+m_{z}^{k}\times \sin r_{k} \end{array}$$

In other words, the three-axis magnetometer can be used as the two-axis magnetometer in horizontal plane coordinate (North-East frame).

### 2.2. Magnetometer Error Source

Similar to inertial sensors such as accelerometers and gyroscopes, magnetometers also suffer from errors due to magnetic deviation which can result in an inaccurate heading measurement [25]. The mainly magnetic deviation can be divided into two categories: hard iron and soft iron distortions [26]. The presence of electro-magnetic systems and ferromagnetic materials beside the magnetometer are the main cause of these errors, that is the host platform itself may be responsible for them [27]. Hard iron distortions are caused by a magnetic source, which produce permanent bias added to each axis of magnetometer output. In other words, magnetic fields generated by different electronic sub-systems in the vicinity of the magnetometer are called hard iron magnetic sources. With the effects of hard iron distortions, the locus of magnetic field intensity will be a center-shift circle. Soft iron distortions are caused by much complex magnetic fields which are generated by ferromagnetic materials. The magnitudes of these magnetic field depend on the incident angle of the Earth’s magnetic field on the material. Hence it changes as the system platform changes its attitude [28]. Soft iron distortions are non-linear and distort the magnetic field both in intensity and direction, making the locus of magnetic field intensity change from circle to ellipse.

### 2.3. Magnetometer Calibration Procedure

Many calibration algorithms have been proposed. One use measurements collected by a 360° rotation of the levelled magnetometer in the horizontal plane to find the maximum and minimum values to estimate biases of the sensor. The iterative least squares algorithm is utilized to estimate the bias and the combined scale factors for a given data set [29]. This algorithm requires a proper uniform distribution of the data over all attitudes. The Constrained Least Squares Ellipsoid Fitting (CLSEF) method utilizes a constrained condition involved with the limited data to achieve an accurate ellipsoid fit [30]. Although these methods have been proved to be effective, they need large amount of computation. Furthermore, the performance of these methods will be degraded when applied to foot-mounted IMU platform, because the floor and shoes can cause interference to the Earth’s magnetic field. To eliminate this interference, we propose an on-board magnetometer calibration approach.

When the foot is stationary on the floor, the platform is only affected by gravity. Then we can calculate the attitude angle of the platform according to Equation (3). Using the attitude angle, we can transform the magnetic field from sensor body coordinate system to navigation coordinate system. In addition, as abovementioned discussion, the orthogonal field component $B_{z}$ can be neglected during the heading estimation procedure, then the three-axis magnetometer is equivalent to the two-axis magnetometer in horizontal plane coordinate. The calibration procedure is described as follows:

#### 2.3.1. Hard Iron Distortion Calibration Procedure

**Figure 2:**
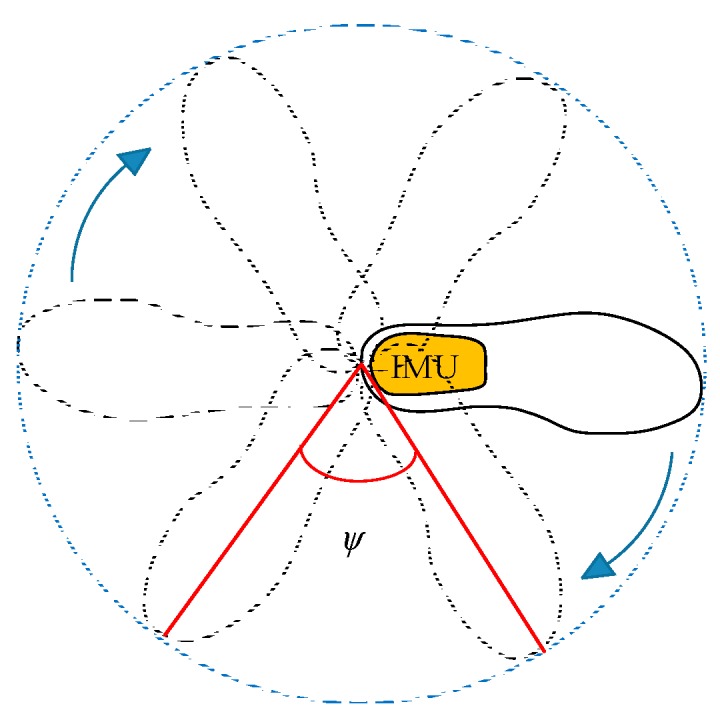
Sketch picture of magnetometer calibration.

(1) Let the IMU be installed in the heel of the pedestrian’s shoe, then the pedestrian walks around a loop as shown in Figure 2. Note that the rotation angle ψ should be as small as possible to store relatively complete measurements of the Earth’s magnetic field. Here, we choose a reference value of ψ from 15° to 35°.
(2) By using the stored data, the stance phase can be identified according to the Zero-Velocity detection algorithm in Reference [31].
(3) Transform the magnetometer’s measurement from the sensor body coordinate frame to the navigation frame according to Equation (4). Then the average maximum and minimum values for each of the axes can be calculated.
(4) Let max(x|y) and min(x|y) denote the maximum and minimum values of x and y axe respectively. Then, determine offsets of each axes as follows:
  (6) $$\begin{array}{l} \mathrm{offset}\_x=(\max(x)+\min(x))/2\\ \mathrm{offset}\_y=(\max(y)+\min(y))/2 \end{array}$$
  where offset_x and offset_y represent the offset of x and y axe respectively. Then these offsets are subtracted from the magnetometer’s data that have been transformed at step (3) to eliminate the hard iron distortion effects.

#### 2.3.2. Soft Iron Distortion Calibration Procedure

Compensation for the soft-iron distortion includes finding mathematical representation of the ellipse that is caused by disturbed magnetic field. The calibration procedure is as follows:

(1) Find major axe and minor axe of the ellipse by a loop calculation of stored magnetic field database M:
  (7) $$r=\sqrt{{\left(m_{x}\right)}^{2}+{\left(m_{y}\right)}^{2}}$$
  where $(m_{x},\;m_{y})\in M$, max(r) is the length of the major axe and min(r) is length of the minor axe. For both max(r) and min(r), we can get corresponding coordinates from M: $\left(m_{x1},\;m_{y}{}_{1}\right)$ and $\left(m_{x2},\;m_{y}{}_{2}\right)$.
(2) Determine inclination angle of the ellipse by:
  (8) $$\eta =\sin^{-1}(m_{y}{}_{1}/\max(r))$$
  Then, the rotational matrix can be constructed to align the rotated ellipse with one of the coordinate system axes. The rotational matrix R is given by:
  (9) $$R=\left[\begin{array}{cc} \cos\;\eta & \sin\;\eta \\ -\sin\;\eta & \cos\;\eta \end{array}\right]\;$$
(3) Rotate the ellipse:
  (10) $$M_{rotated}=R(\eta )\times M$$
  where $M_{rotated}$ represents the set of coordinates after rotated. To scale the two-axis length of ellipse equal, the scale factor is required τ=min(r)/max(r).
(4) Scale X axe (or Y axe) coordinate of the ellipse to make it circular:
  (11) $$M_{rotated}^{\prime }(X)=M_{rotated}(X)\cdot \tau \;$$
  The coordinates set of the circle is rotated back to initial position using the rotational matrix and inclination angle:
  (12) $$M_{corrected}=R(-\eta )\times M_{rotated}^{\prime }$$
  where $M_{corrected}$ represents the final result of the magnetometer calibration.

## 3. Quasi-Static Magnetic Field Detection

**Figure 3:**
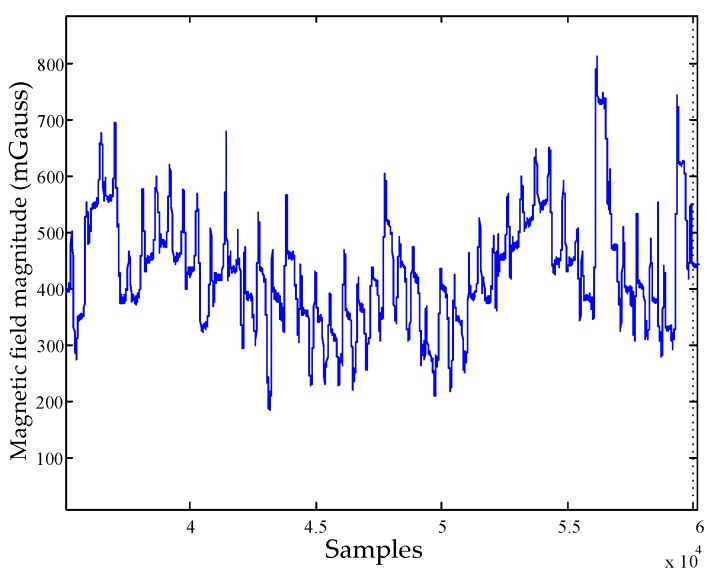
The magnitude of the total magnetic field in indoor environment.

Although the Earth’s magnetic field is a good measurement for heading estimation outdoor, it suffers severe perturbations in buildings. These perturbations induce random variations in the magnetic field estimations. As Figure 3 shows, the magnetic field magnitude in indoor environments is not constant due to the changing perturbations. Although the magnetic field indoor is not spatially constant, depending on the surroundings, it is possible to have locations as well as short periods when the perturbed magnetic field is constant which can be considered quasi-static [23]. The QSF method detects quasi-static magnetic field based on the change rate of total magnetic field magnitude. This method causes false-alarm detection easily when the magnitude of each axis changes with different ratio but the total magnetic field magnitude has very slight changes. The iQSF method adds two more detection constraints including the change rate of headings derived from magnetic field and angular rates (gyroscope output). The iQSF method can only take effect when the pedestrian walks along straight-line paths. If the pedestrian moves along non-ideal paths (curved or more complex paths), the change rate of the heading will be far greater than zero and the quasi-static magnetic field will be missing.

To solve this problem, we use the rate of change in difference between the two headings mentioned above. As the error accumulation of the heading derived from gyroscope is very small during short periods, the heading derived from angular rate can be utilized to assess the stability of the magnetically derived heading. If the rate of change in differences between the magnetically derived heading and the heading derived from angular rate is approximate to zero, then the magnetic field can be considered as quasi-static. Herein, the detection of the quasi-static magnetic field is unrelated to the shape of the paths. In addition, the measurements of total magnetic field magnitude and inclination angle are also adopted. The magnetic field can be detected as quasi-static if both the changing rates of total magnetic field magnitude and inclination angle are equal to zero. The proposed detection approach is elaborated as follows.

Let $\theta_{k}^{m}$ be the magnetically derived heading at the $k^{th}$ step, $\theta_{k}^{g}$ represents that derived from the angular rate. $\left\|B_{k}\right\|$ denotes the magnitude of the magnetic field, $I_{k}$ denotes the inclination angle. The model of the measurement is as follows:

(13) $$y_{k}=s_{k}+v_{k}$$

where $s_{k}={\left[\begin{array}{ccc} s_{k}^{\theta } & s_{k}^{\varphi } & s_{k}^{B} \end{array}\right]}^{T}$ and T denotes the transpose operation. Moreover, $s_{k}^{\theta }=\left|\theta_{k}^{m}-\theta_{k}^{g}\right|$ denotes the difference between the magnetically derived heading and the heading derived from angular rate at the $k^{th}$ step, $s_{k}^{I}=\left|I_{k}-I_{k-1}\right|$ denote change in the inclination angle of two adjacent steps; $s_{k}^{B}=\left|\left\|B_{k}\right\|-\left\|B_{k-1}\right\|\right|$ represents change in the magnitude of the magnetic field. $v_{k}={\left[\begin{array}{ccc} v_{k}^{\theta } & v_{k}^{I} & v_{k}^{B} \end{array}\right]}^{T}$ denotes the measurement noise and we assume that the noise is independent identically distributed zero-mean, Gaussian with covariance matrix:

(14) $$C_{v}=Ε\{v_{k}v_{k}^{T}\}=\left[\begin{array}{ccc} \sigma_{\theta }^{2} & 0 & 0\\ 0 & \sigma_{I}^{2} & 0\\ 0 & 0 & \sigma_{B}^{2} \end{array}\right]$$

where $\sigma_{\theta }^{2}$, $\sigma_{I}^{2}$ and $\sigma_{B}^{2}$ are the noise variance of $s_{k}^{\theta }$, $s_{k}^{I}$ and $s_{k}^{B}$, respectively. Ε{⋅} denotes the expectation operation. Mathematically, the magnetic field can be detected as a quasi-static field if $s_{k}^{\theta }=0$, $s_{k}^{I}=0$ and $s_{k}^{B}=0$. Then the detection problem can be formalized as a binary hypothesis testing problem and the detector can choose between the two hypotheses $H_{0}$ or $H_{1}$ defined as follows:

(15) $$\begin{array}{l} H_{0}:\exists k\in \Gamma_{n}s.t.\begin{array}{ccccc} s_{k}^{\theta }\neq 0 & \mathrm{or} & s_{k}^{I}\neq 0 & \mathrm{or} & s_{k}^{B}\neq 0 \end{array}\\ H_{1}:\forall k\in \Gamma_{n}\begin{array}{c} \mathrm{then} \end{array}\begin{array}{c} \begin{array}{ccccc} s_{k}^{\theta }=0 & \mathrm{and} & s_{k}^{I}=0 & \mathrm{and} & s_{k}^{B}=0 \end{array} \end{array} \end{array}$$

where the hypothesis $H_{0}$, $H_{1}$ denote non-static field and quasi-static field, respectively. $\Gamma_{n}$ denotes the step interval and $\Gamma_{n}=\left\{\ell \in N:\;n\leq \ell \leq n+N-1\right\}$. The performance of the detector is specified by the false-alarm probability $P_{FA}$ and detection probability $P_{D}$. Neyman–Pearson theorem tells us how to choose between the two hypotheses to maximize $P_{D}$ for a given $P_{FA}$. Based on the measurement model specified by Equation (13), the PDFs for the hypothesis $H_{i}\;(i=0,1)$ is given by:

(16) $$\begin{array}{ll} p\left(z_{n};\;s_{k},\;H_{i}\right) & =\prod_{k\in \Gamma_{n}}p\left(y_{k};\;s_{k},\;H_{i}\right)\\ & =\prod_{k\in \Gamma_{n}}p\left(y_{k}^{\theta };\;s_{k}^{\theta },\;H_{i}\right)p\left(y_{k}^{I};\;s_{k}^{I},\;H_{i}\right)p\left(y_{k}^{B};\;s_{k}^{B},\;H_{i}\right) \end{array}$$

where

(17) $$\begin{array}{l} p\left(y_{k}^{\theta };\;s_{k}^{\theta },\;H_{i}\right)=\frac{1}{{\left(2\pi \sigma_{\theta }^{2}\right)}^{3/2}}\exp\left(-\frac{1}{2\sigma_{\theta }^{2}}{\left(y_{k}^{\theta }-s_{k}^{\theta }\right)}^{2}\right)\\ p\left(y_{k}^{I};\;s_{k}^{I},\;H_{i}\right)=\frac{1}{{\left(2\pi \sigma_{I}^{2}\right)}^{3/2}}\exp\left(-\frac{1}{2\sigma_{\varphi }^{2}}{\left(y_{k}^{I}-s_{k}^{I}\right)}^{2}\right)\\ p\left(y_{k}^{B};\;s_{k}^{B},\;H_{i}\right)=\frac{1}{{\left(2\pi \sigma_{B}^{2}\right)}^{3/2}}\exp\left(-\frac{1}{2\sigma_{B}^{2}}{\left(y_{k}^{B}-s_{k}^{B}\right)}^{2}\right) \end{array}$$

$z_{n}$ denotes the measurement sequence and $z_{n}≜{\left\{y_{k}\right\}}_{k=n}^{n+N-1}$. Under the hypothesis $H_{1}$, $s_{k}^{\theta }=0$, $s_{k}^{I}=0$ and $s_{k}^{B}=0$, hence the PDF for $H_{1}$ is given by

(18) $$\begin{array}{ll} p\left(z_{n};\;s_{k},\;H_{1}\right) & =\frac{1}{{\left(2\pi \sigma_{\theta }^{2}\right)}^{3N/2}}\exp\left(-\frac{1}{2\sigma_{\theta }^{2}}\sum_{k\in \Gamma_{n}}{\left(y_{k}^{\theta }\right)}^{2}\right)\\ & \cdot \frac{1}{{\left(2\pi \sigma_{I}^{2}\right)}^{3N/2}}\exp\left(-\frac{1}{2\sigma_{I}^{2}}\sum_{k\in \Gamma_{n}}{\left(y_{k}^{I}\right)}^{2}\right)\\ & \cdot \frac{1}{{\left(2\pi \sigma_{B}^{2}\right)}^{3N/2}}\exp\left(-\frac{1}{2\sigma_{B}^{2}}\sum_{k\in \Gamma_{n}}{\left(y_{k}^{B}\right)}^{2}\right) \end{array}$$

Owing to the lack of knowledge about the signal $s_{k}$, we cannot completely specify the PDFs under the hypothesis $H_{0}$ and apply the Likelihood Ratio Test (LRT) derived from Neyman–Pearson theorem. However, by replacing the unknown signal $s_{k}$ with its Maximum Likelihood Estimates (MLE), we can derive a Generalized Likelihood Ratio Test (GLRT). The MLE is obtained by maximizing Equation (16) with respect to the unknown parameters. Under the hypothesis $H_{0}$, the MLE ${\hat{s}}_{k}$ of the unknown $s_{k}$ can be obtained depending on the theory of MLE for vector parameters as follows:

(19) $${\frac{\partial \ln p\left(z_{n};\;s_{k},\;H_{0}\right)}{\partial s_{k}}|}_{s_{k}={\hat{s}}_{k}}=0$$

Then we can get:

(20) $${\hat{s}}_{k}^{\theta }=\frac{1}{N}\sum_{k\in \Gamma_{n}}y_{k}^{\theta }，{\hat{s}}_{k}^{I}=\frac{1}{N}\sum_{k\in \Gamma_{n}}y_{k}^{I},{\hat{s}}_{k}^{B}=\frac{1}{N}\sum_{k\in \Gamma_{n}}y_{k}^{B}$$

Substituting ${\hat{s}}_{k}$ into Equation (16) yields

(21) $$\begin{array}{ll} p\left(z_{n};\;{\hat{s}}_{k},\;H_{0}\right) & =\frac{1}{{\left(2\pi \sigma_{\theta }^{2}\right)}^{3N/2}}\exp\left(-\frac{1}{2\sigma_{\theta }^{2}}\sum_{k\in \Gamma_{n}}{\left(y_{k}^{\theta }-\frac{1}{N}\sum_{k\in \Gamma_{n}}y_{k}^{\theta }\right)}^{2}\right)\\ & \cdot \frac{1}{{\left(2\pi \sigma_{I}^{2}\right)}^{3N/2}}\exp\left(-\frac{1}{2\sigma_{I}^{2}}\sum_{k\in \Gamma_{n}}{\left(y_{k}^{I}-\frac{1}{N}\sum_{k\in \Gamma_{n}}y_{k}^{I}\right)}^{2}\right)\\ & \cdot \frac{1}{{\left(2\pi \sigma_{B}^{2}\right)}^{3N/2}}\exp\left(-\frac{1}{2\sigma_{B}^{2}}\sum_{k\in \Gamma_{n}}{\left(y_{k}^{B}-\frac{1}{N}\sum_{k\in \Gamma_{n}}y_{k}^{B}\right)}^{2}\right) \end{array}$$

The GLRT decides on $H_{1}$, if

(22) $$T_{G}=\frac{p\left(z_{n};\;s_{k},\;H_{1}\right)}{p\left(z_{n};\;{\hat{s}}_{k},\;H_{0}\right)}\;>\;\gamma$$

where the threshold *γ* is determined from

(23) $$P_{FA}=\int_{\left\{z_{n}:T_{G}>\gamma \right\}}p\left(z_{n};\;{\hat{s}}_{k},\;H_{0}\right)dz_{n}=\alpha \;$$

If we combine Equations (18), (21) and (22), $T_{G}$ can be described as

(24) $$\begin{array}{ll} T_{G} & =\exp\left(-\frac{1}{2\sigma_{\theta }^{2}}\sum_{k\in \Gamma_{n}}\left[{\left(y_{k}^{\theta }\right)}^{2}-{\left(y_{k}^{\theta }-\frac{1}{N}\sum_{k\in \Gamma_{n}}y_{k}^{\theta }\right)}^{2}\right]\right)\\ & \cdot \exp\left(-\frac{1}{2\sigma_{I}^{2}}\sum_{k\in \Gamma_{n}}\left[{\left(y_{k}^{I}\right)}^{2}-{\left(y_{k}^{I}-\frac{1}{N}\sum_{k\in \Gamma_{n}}y_{k}^{I}\right)}^{2}\right]\right)\\ & \cdot \exp\left(-\frac{1}{2\sigma_{B}^{2}}\sum_{k\in \Gamma_{n}}\left[{\left(y_{k}^{B}\right)}^{2}-{\left(y_{k}^{B}-\frac{1}{N}\sum_{k\in \Gamma_{n}}y_{k}^{B}\right)}^{2}\right]\right) \end{array}$$

Simplifying yields

(25) $$T_{G}=\exp\left(-\frac{1}{2N\sigma_{\theta }^{2}}(\sum_{k\in \Gamma_{n}}y_{k}^{\theta }{)}^{2}\right)\times \exp\left(-\frac{1}{2N\sigma_{I}^{2}}(\sum_{k\in \Gamma_{n}}y_{k}^{I}{)}^{2}\right)\times \exp\left(-\frac{1}{2N\sigma_{B}^{2}}(\sum_{k\in \Gamma_{n}}y_{k}^{B}{)}^{2}\right)$$

Taking the natural logarithm on both sides of Equation (22) and simplifying yields:

(26) $$\frac{1}{N}\left(\frac{1}{\sigma_{\theta }^{2}}(\sum_{k\in \Gamma_{n}}y_{k}^{\theta }{)}^{2}+\frac{1}{\sigma_{I}^{2}}(\sum_{k\in \Gamma_{n}}y_{k}^{I}{)}^{2}+\frac{1}{\sigma_{B}^{2}}(\sum_{k\in \Gamma_{n}}y_{k}^{B}{)}^{2}\right)\;<\;\gamma^{\prime }$$

where $\gamma^{\prime }=-2\ln\gamma$. If the inequality in Equation (26) is satisfied, then the GLRT chooses the hypothesis that the magnetic field is quasi-static.

## 4. Heading Error Estimation 

If the magnetic field is detected as quasi-static, the magnetically derived heading will be adopted to estimate the heading errors of the gyroscope based on the ZUPT-aided EKF algorithm. Let $\omega_{t}$ be three-axis gyroscope output at time t, $C_{t-1}$ denotes the rotation matrix that transforms from the body to the navigation frame at time t−1, which is updated with the gyroscopic information. A *Padé* approximation of the exponential function is utilized to update orientation [18]:

(27) $$C_{t}=f(C_{t-1},\;\omega_{t})=C_{t-1}\times \frac{2I_{3}+\Omega_{t}\times \Delta t}{2I_{3}-\Omega_{t}\times \Delta t}$$

where Δt denotes the sample interval, $I_{3}$ represents the unit matrix of three order, $\Omega_{t}$ is the skew symmetric matrix of angular rates:

(28) $$\Omega_{t}=\left[\begin{array}{ccc} 0 & -\omega_{t}(3) & \omega_{t}(2)\\ \omega_{t}(3) & 0 & -\omega_{t}(1)\\ -\omega_{t}(2) & \omega_{t}(1) & 0 \end{array}\right]$$

Let $\delta x_{t|t}$ be the error state vector of EKF at time t:

(29) $$\delta x_{t|t}={\left[\delta \phi_{t},\;\delta \omega_{t},\;\delta r_{t},\;\delta v_{t},\;\delta a_{t}\right]}^{T}$$

The vector $\delta x_{t|t}$ contains 5 components, namely the estimated errors in attitude ($\delta \phi_{t}$), positions ($\delta r_{t}$), velocity ($\delta v_{t}$), the bias of the gyroscope ($\delta \omega_{t}$) and the accelerometer ($\delta a_{t}$). All these 5 components have three elements each, corresponding to the three-dimension estimation. The linearized state transition model is:

(30) $$\delta x_{t|t-1}=\Phi_{t}\delta x_{t-1|t-1}+w_{t-1}$$

where $\delta x_{t|t-1}$ is the one step predicted error state, $\delta x_{t-1|t-1}$ is the last filtered error state, $w_{t-1}$ is the process noise with the covariance matrix $Q_{t}=E(w_{t}w_{t}^{T})$. $\Phi_{t}$ is the 15×15 state transition matrix:

(31) $$\Phi_{t}=\left[\begin{array}{ccccc} I_{3} & \Delta t\cdot C_{t} & 0_{3} & 0_{3} & 0_{3}\\ 0_{3} & I_{3} & 0_{3} & 0_{3} & 0_{3}\\ 0_{3} & 0_{3} & I_{3} & \Delta t\cdot I_{3} & 0_{3}\\ -\Delta t\cdot S(a_{t}) & 0_{3} & 0_{3} & I_{3} & \Delta t\cdot C_{t}\\ 0_{3} & 0_{3} & 0_{3} & 0_{3} & I_{3} \end{array}\right]$$

where $0_{3}$ denotes 3×3 zero matrix and $S(a_{t})$ is the skew symmetric matrix for accelerations that allows the EKF to act as an inclinometer

(32) $$S(a_{t})=\left[\begin{array}{ccc} 0 & -a_{t}^{z} & a_{y}^{z}\\ a_{t}^{z} & 0 & -a_{t}^{z}\\ -a_{y}^{z} & a_{t}^{z} & 0 \end{array}\right]$$

$a_{t}=[a_{t}^{x},a_{t}^{y},a_{t}^{z}]$ is the acceleration that has been transformed to the navigation frame. The measurement model is

(33) $$z_{t}=H\delta x_{t|t}+n_{t}$$

where $z_{t}$ is the error measurements, H is the measurement matrix, $n_{t}$ is the measurement noise with the covariance matrix $G_{t}=E(n_{t}n_{t}^{T})$. If the magnetic field is detected as quasi-static during the stance phase, the error measurement $m_{t}$ can be calculated as:

(34) $$m_{t}=[\Delta \theta_{t},\;v_{t}]$$

where $v_{t}$ is the velocity error derived from the accelerometer. $\Delta \theta_{t}$ is the heading error derived from the gyroscope:

(35) $$\Delta \theta_{t}=\theta_{t}^{g}-\theta_{t}^{m}$$

Here, as the magnetic field is detected as quasi-static, the heading $\theta_{t}^{m}$ derived from the magnetic field can be utilized as a reliable measurement to eliminate heading errors of the gyroscope. The measurement matrix H, for ZUPT update, is given by:

(36) $$H=\left[\begin{array}{c} 0_{3},I_{3},0_{3},0_{3},0_{3}\\ 0_{3},0_{3},0_{3},I_{3},0_{3} \end{array}\right]$$

Then the filtered error state $\delta x_{t|t}$ is obtained:

(37) $$\delta x_{t|t}=\delta x_{t|t-1}+K_{t}\cdot [m_{t}-H\delta x_{t|t-1}]$$

where $K_{t}$ is the Kalman gain matrix. The attitude refinement is achieved by updating the rotation matrix $C_{t}$ with the attitude errors $\delta \phi_{t}$. Assuming $\delta \phi_{t}$ is small, the corrected rotation matrix $C_{t}^{\prime }$ is given by:

(38) $$C_{t}^{\prime }=g(C_{t},\;\delta \phi_{t})=\frac{2I_{3}+\Theta_{t}\times \Delta t}{2I_{3}-\Theta_{t}\times \Delta t}\times C_{t}$$

where $\Theta_{t}$ is the skew symmetric matrix for small angles:

(39) $$\Theta_{t}=\left[\begin{array}{ccc} 0 & -\delta \phi_{t}(3) & \delta \phi_{t}(2)\\ \delta \phi_{t}(3) & 0 & -\delta \phi_{t}(1)\\ -\delta \phi_{t}(2) & \delta \phi_{t}(1) & 0 \end{array}\right]$$

**Figure 4:**
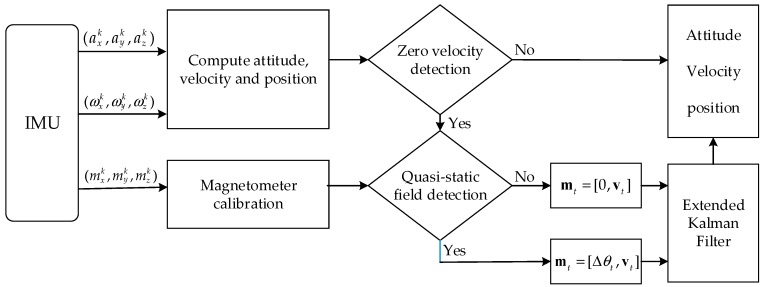
Flowchart of the proposed method.

According to the above description, we summarize the procedure of the proposed method and the flowchart is shown in Figure 4.

## 5. Results and Discussion

**Figure 5:**
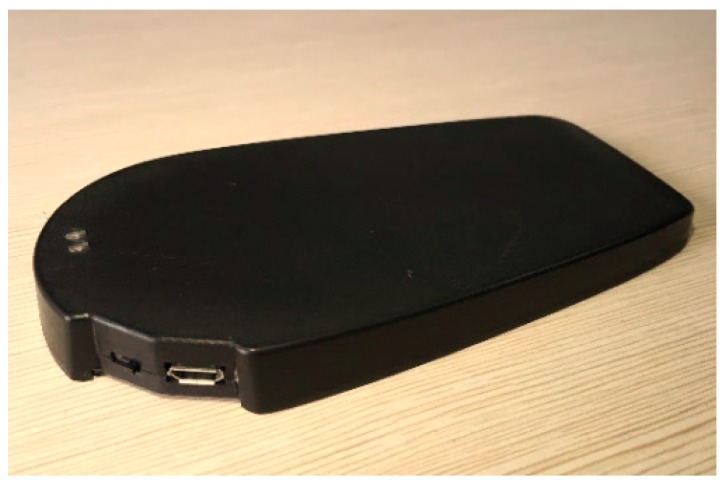
The insole-shaped MIMU module.

To analyze the performance of the proposed method, experiments have been carried out. As shown in Figure 5, the foot-mounted module we use in these experiments is a Multiple Inertial Measurement Units (MIMU) platform. The platform holds 8 MPU9250 IMUs (InvenSense Inc., San Jose, CA, USA), with 4 on the top side and 4 on the bottom side. The data is stored and can be transferred to a computer via a USB interface. Each MPU9250 IMU consists of a three-axis MEMS accelerometer, a three-axis MEMS gyroscope and a three-axis MEMS magnetometer. The advantage of the MIMU platform has already been elaborated in Reference [32], so it will not be discussed here. The system is mounted in the heel of shoes and the sampling rate is 400 Hz.

### 5.1. Magnetometer Calibration Experiments

**Figure 6:**
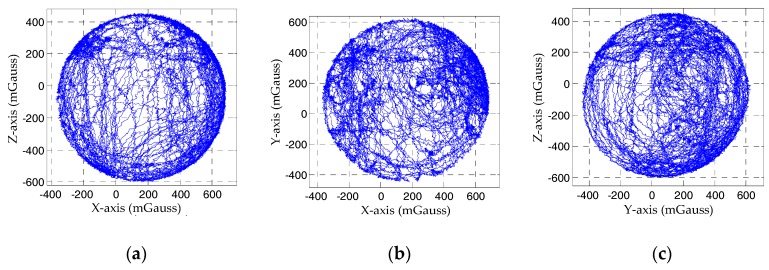
The magnetic field measurements of rotation: (**a**) Magnetic field measurements of *X-Z* plane; (**b**) Magnetic field measurements of *X-Y* plane; (**c**) Magnetic field measurements of *Y-Z* plane.

We conducted one test to assess the performance of the proposed magnetometer calibration method. In the test, the proposed method was compared with the CLSEF method. We performed the CLSEF method by rotating the sensor in all possible directions in a constant uniform magnetic field. The sensor measurement of rotation is depicted in Figure 6 and the calibration parameters of the two methods are summarized in Table 1. The variable $K_{c}$ denotes the combination of the soft iron errors, misalignment and scale factor errors and $B_{c}$ represent hard iron errors.

**Figure 7:**
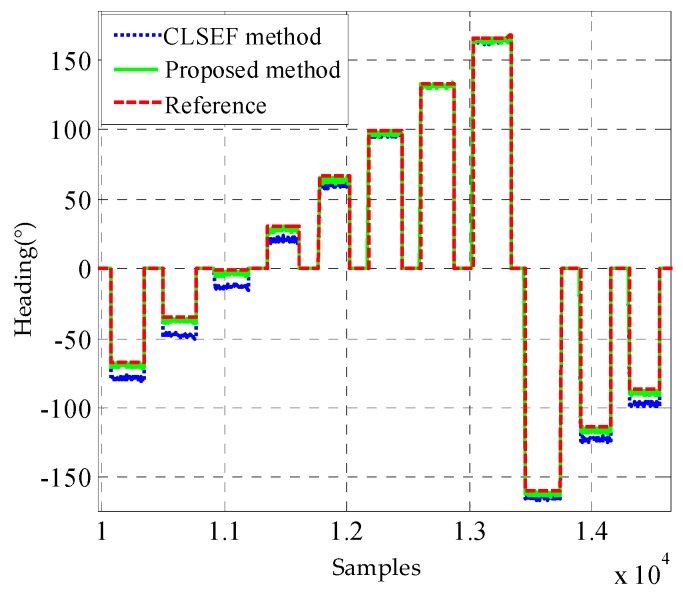
The heading calibration comparison between the CLSEF method and the proposed method.

The results for heading calibration by the two methods are compared in Figure 7. As the drift of gyroscope is very slow, it can be ignored during the magnetometer calibration procedure. Therefore, the heading derived from gyroscopes can be utilized as the reference to assess the performance of the two methods. In Figure 7, the difference between the reference and the proposed is no more than 5° and much smaller than the CLSEF method. Although the CLSEF calibration method can provide accuracy calibration parameters in free space scenario, its performance will be degraded in the foot-mounted application because of the effects caused by the floor and shoes. The proposed calibration method can eliminate most of the effects and performs better in the foot-mounted application scenario.

### 5.2. Heading Error Correction Experiments

**Figure 8:**
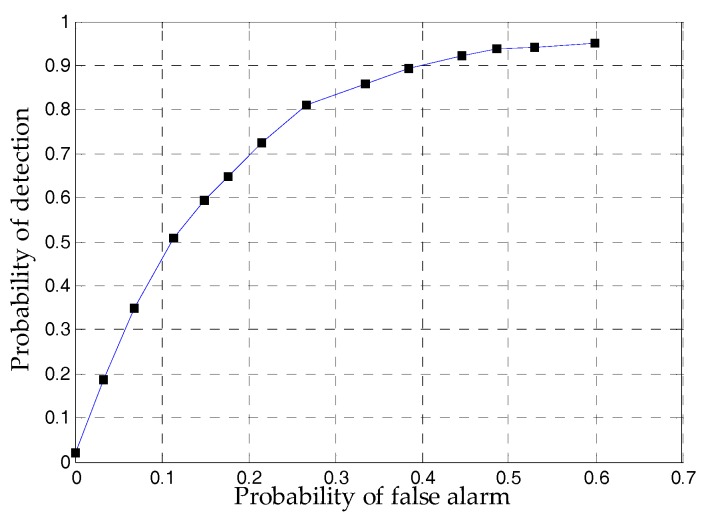
The ROC curve of the proposed detector.

To use the proposed detector effectively, there are several tuning parameters, denoted in Equation (26), need to be quantified.$\sigma_{\theta }$, $\sigma_{I}$ and $\sigma_{B}$ give a measure of the noise which is encountered during quasi-static field periods. To evaluate these parameters, the magnetometer and gyroscope data has been collected for more than two hours. The corresponding three kinds of noise variance are measured as $\sigma_{\theta }=0.067\;\mathrm{rad}/s$, $\sigma_{I}=0.054\;\mathrm{rad}/s$ and $\sigma_{B}=5.35\;\mathrm{mGs}$. The threshold $\gamma^{\prime }$ can be selected based on the relationship between the probability of detection and the acceptable probability of false alarms. The Receiver Operating Characteristics (ROC) curve is utilized for this and it also defines the performance of the detector. The ROC of the detectors was calculated by varying the thresholds of the detectors and comparing the decisions of the detectors to the output of the reference system. We calculated the test statistics for the detectors using a window size N=3. Figure 8 shows the ROC curve of the proposed detector. When test threshold is set to 170, the corresponding probability of detection is about 0.8. When the probability of detection is larger than 0.8, the false alarm probability increases much faster than the detection probability. Thus, we choose 170 as the test threshold.

**Figure 9:**
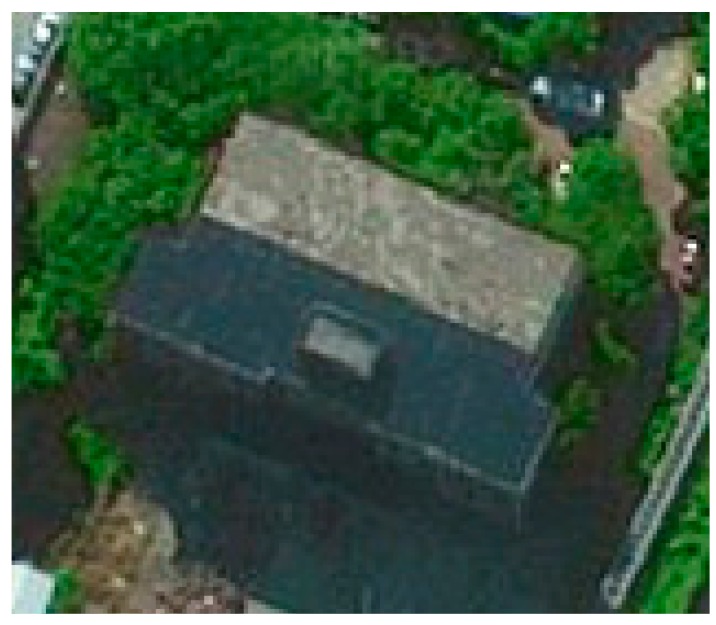
The bird’s eye view of the office test environment.

To evaluate the positioning performance of the proposed method, we conducted two tests in real indoor environments which are very common for pedestrian navigation application: office and shopping mall. In the office test, we walked along the corridors and walls in a typical office at normal speed (approximately 5 km/h) and then kept walking three loops with the same path. The total distance of the path was approximately 1018 m in 786 steps. Figure 9 gives the bird’s eye view of the test region selected in the school where the authors work.

**Figure 10:**
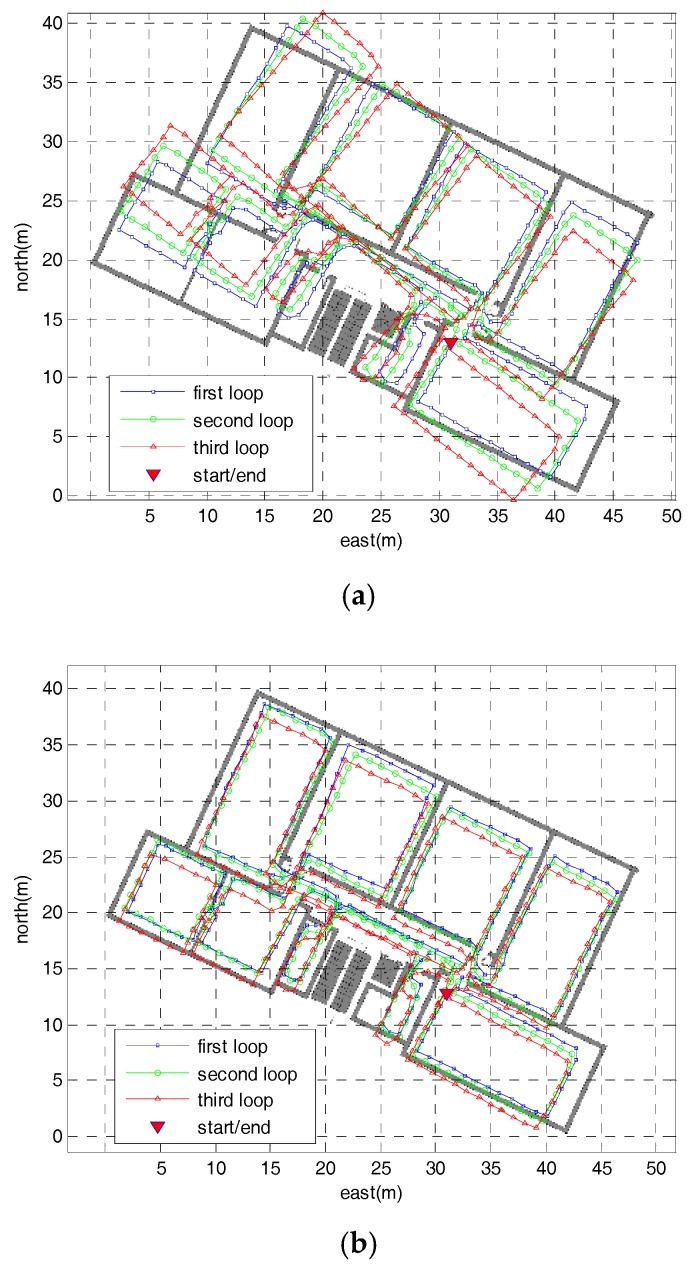

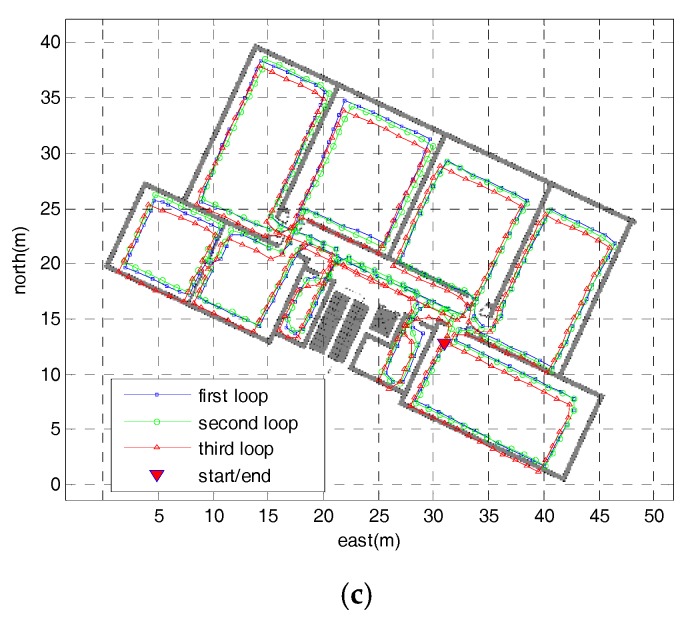
The step-wise trajectories of the office test: (**a**) Step-wise trajectory of the pure ZUPT method; (**b**) Step-wise trajectory of the iQSF method; (**c**) Step-wise trajectory of the proposed method.

Figure 10 depicts the step-wise trajectories obtained using the pure ZUPT, iQSF methods and the proposed method, respectively. In Figure 10a the heading error accumulates due to gyro biases which cannot be observed using the pure ZUPT. The final positioning errors and heading errors (difference between the initial and final heading) reach up to 2 m and 14° respectively. The iQSF method and the proposed method perform much better than the pure ZUPT algorithm as shown in Figure 10b,c. Both methods achieve positioning error of no more than 0.5 m. As most of the paths are straight lines in this test, so the iQSF method can also detect most potential quasi-static magnetic field and then estimates the heading errors of the gyroscope.

**Figure 11:**
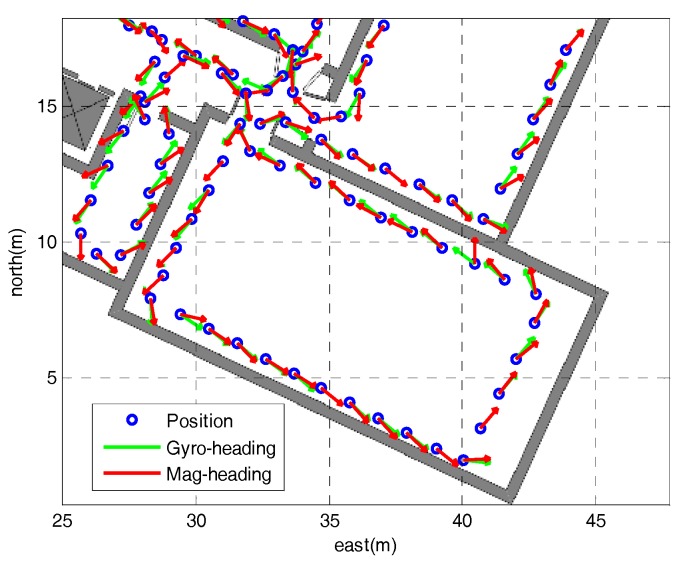
The heading results of the first loop using the proposed method.

Figure 11 shows the heading results of the first loop using the proposed method. It can be seen that although the magnetically derived heading sometimes changes violently due to the perturbations, there is the moment when it is stable and similar to the heading derived from gyroscope, which can be used to estimate the heading error originating from the gyroscope drift.

**Figure 12:**
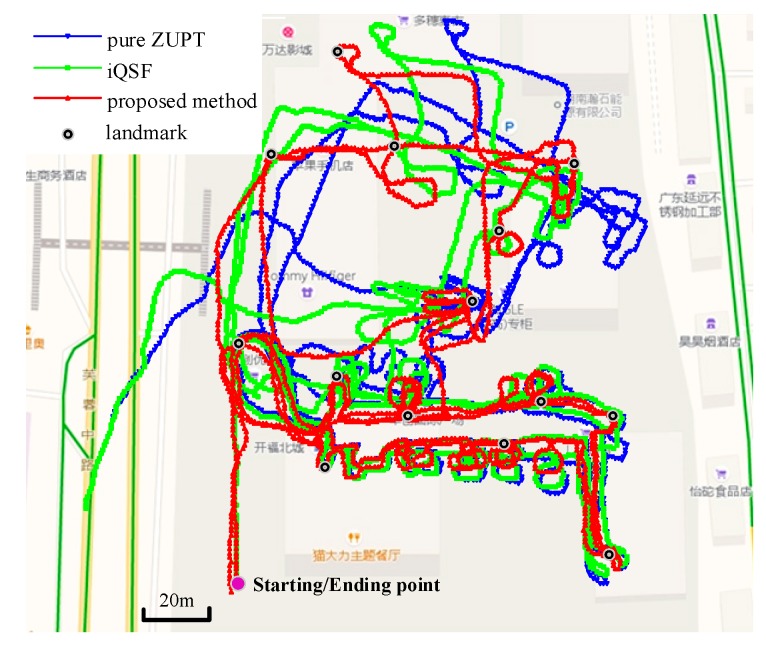
Trajectories obtained using pure ZUPT, iQSF and the proposed method.

We conducted the second trajectory test in a shopping mall to evaluate the effectiveness of the proposed method in large-scale scenario. The total distance of this trajectory was approximately 2480 m in 1920 steps and 14 landmarks were set along the path to calculate the positioning errors. Figure 12 shows the trajectories obtained using pure ZUPT, iQSF and the proposed method. The maximum trajectory error obtained using the iQSF method is approximately 51 m whereas that for the proposed method is approximately 3 m.

**Figure 13:**
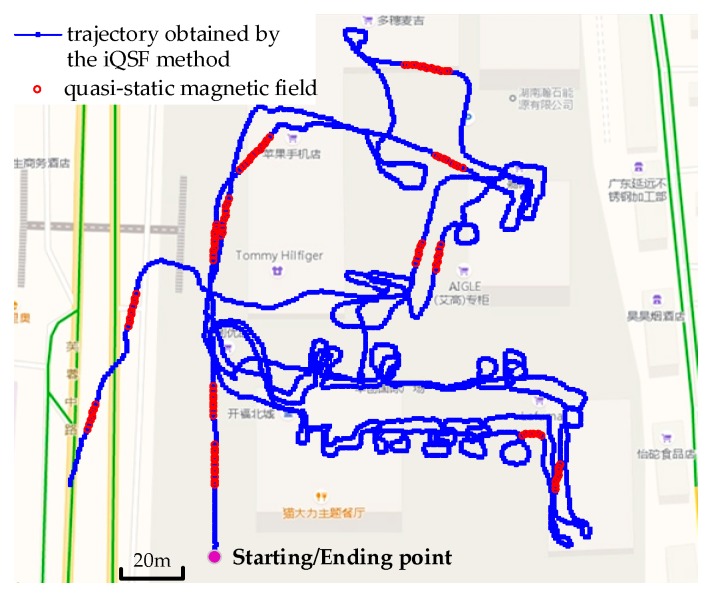
The detection results of quasi-static magnetic field using iQSF method.

**Figure 14:**
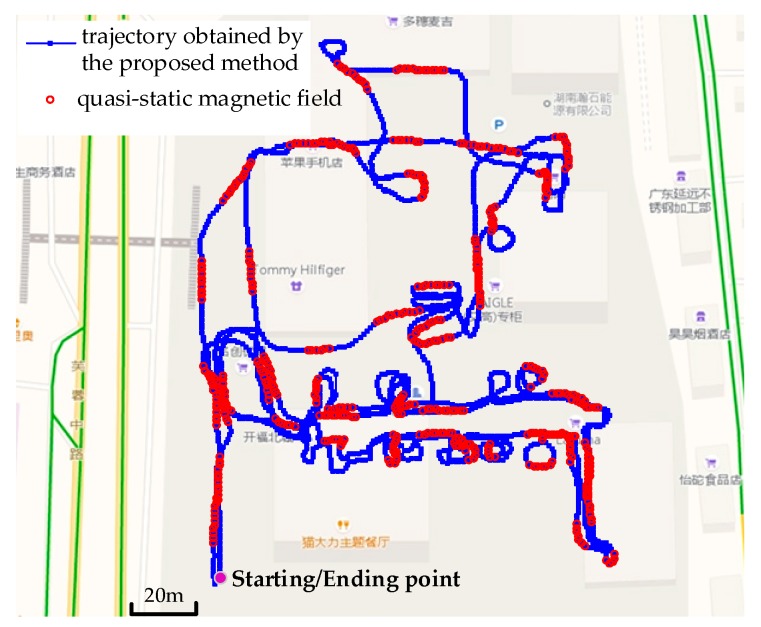
The detection results of quasi-static magnetic field of the proposed method.

Figure 13 and Figure 14 depict the detection results of quasi-static magnetic field using iQSF and the proposed method, respectively. Compared with the office test, the trajectory of the shopping mall test is non-straight for most of the time. In this case, most of the potential quasi-static magnetic field cannot be detected using the iQSF method, while the proposed method is not subject to the straight line constraint. The quasi-static magnetic field can be detected successfully using the proposed method and then the gyroscope biases are estimated and compensated during the quasi-static magnetic field periods.

**Figure 15:**
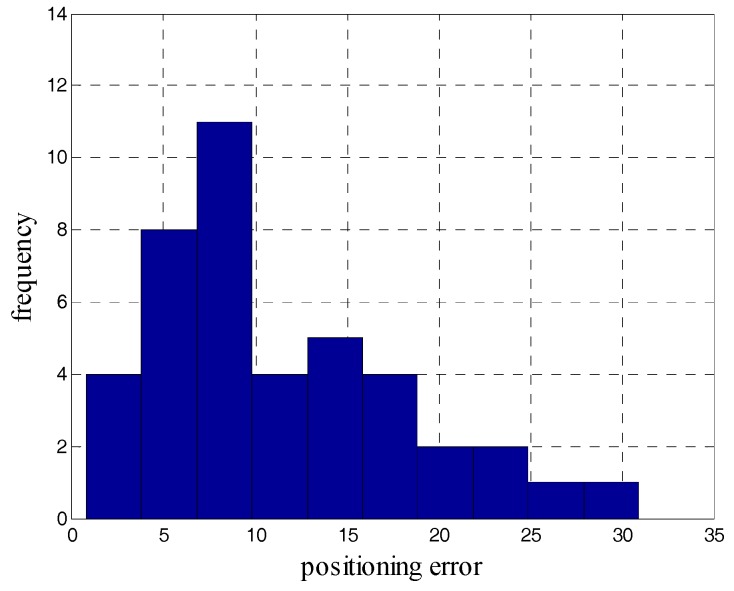
The positioning errors of the iQSF method.

**Figure 16:**
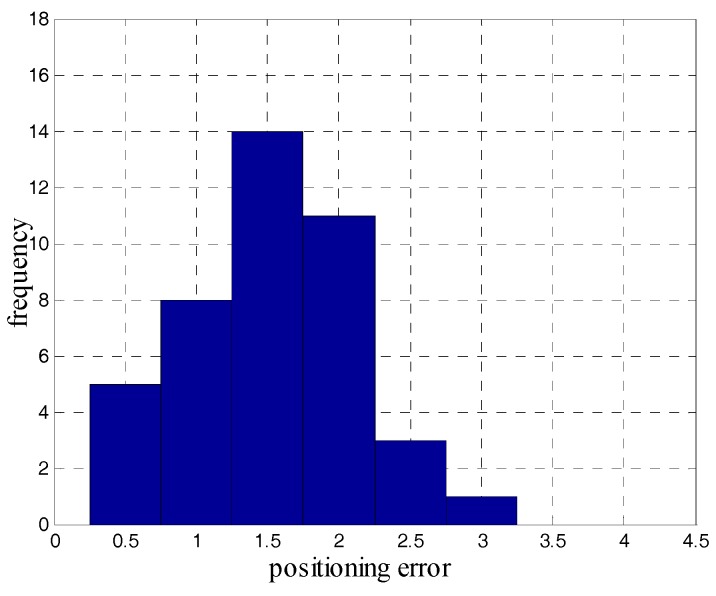
The positioning errors of the proposed method.

During the test, we crossed the 14 landmarks for a total of 42 times and counted the positioning errors of the iQSF method and the proposed method as shown in Figure 15 and Figure 16. The mean error trajectory obtained using the iQSF method has a mean positioning error of 11.3 m as compared to 1.5 m in case of the proposed method. Thus, the positioning errors are reduced by 85% by utilizing the proposed method for constraining the heading drift. It is worth mentioning here that all the test results are concluded from the proposed method without any aiding maps or other external signals.

## 6. Conclusion

In this paper, a heading error estimation method for using the quasi-static magnetic field has been developed. The proposed approach is implemented in two sections. Firstly, to eliminate the errors originating from the system platform, floor and shoes, a magnetometer calibration method is proposed. In the proposed calibration method, the three-axis magnetometer calibration is transformed to that of two-axis according to the motion model of the pedestrian, hence the calibration procedure is simplified. Secondly, a novel quasi-static magnetic field detection approach is developed and utilized to suppress the heading drift of the gyroscope. The proposed method detects the quasi-static magnetic field using the change rate of the magnetic field magnitude, the inclination angle and the difference between the magnetically derived heading and the heading derived from gyroscope. The detected quasi-static magnetic field is utilized as the measurement fed into the ZUPT-aided EKF to estimate the heading errors. 

Selection of two real indoor environments (office and shopping mall) provided data sets for a detailed analysis of the performance of the proposed method. The experimental results indicate that the proposed method is capable of effectively suppressing the heading errors caused by the drift of gyroscopes, reducing the overall positioning error budget by over 85% in shopping mall environment. No other sensors of any kind are used in the proposed method, except IMUs and magnetometers. The approach developed herein are self-contained and assumed a denied GNSS environment. However, GNSS is partly available in numerous indoor environments and urban canyons. Therefore, we will integrate the two approaches to maximize availability and accuracy in the future.

## Figures and Tables

**Table 1:** Summary of the calibration parameters from the magnetometer calibration test.

**Proposed Method**	offset_x **(mGs)**	offset_y **(mGs)**	η **(rad)**	τ	**R**
	137.95	75.26	0.973	0.896	$\left[\begin{array}{cc} 0.562 & 0.827\\ -0.827 & 0.562 \end{array}\right]$
**Clsef** **Method**	$K_{c}$			$B_{c}$	
	$\left[\begin{array}{ccc} 1.027 & 0.037 & 0.046\\ 0 & 1.018 & -0.002\\ 0 & 0 & 1.062 \end{array}\right]$			$\left[\begin{array}{c} 143.36\\ 79.75\\ -56.41 \end{array}\right]$	
